# Analysis of prefabricated myofunctional appliances with different overjet and bumper designs: a three-dimensional finite element analysis

**DOI:** 10.1186/s12903-024-04325-3

**Published:** 2024-05-14

**Authors:** Wu Xiaowei, Lv Haoran, Chen Xuehui, Pan Xiaogang

**Affiliations:** Department of Orthodontics, Shanghai Ninth People’s Hospital, College of Stomatology, National Clinical Research Center for Oral Diseases, Shanghai Key Laboratory of Stomatology & Shanghai Research Institute of Stomatology, Shanghai Jiao Tong University School of Medicine, No. 639 Zhizaoju Road, Huangpu District, Shanghai, 200011 PR China

**Keywords:** Early treatment, Prefabricated myofunctional appliances, Shape deformation, Orthodontics

## Abstract

**Background:**

Prefabricated myofunctional appliance can guide tooth eruption, improve dentition alignment, correct myofunctional disorders and harmful oral habits. However, its application to skeletal discrepancy may result in unsatisfactory tooth inclination. This study aimed to construct a novel appliance with overjet design to avoid this side effect and investigated its shape and mechanical changes under occlusion using three-dimensional finite element method.

**Methods:**

We established three samples of prefabricated myofunctional appliances. The first one was edge to edge without overjet, and the outer shield of both jaws were flattened. The second one was 3 mm overjet with stepped the outer shield. The last one was 3 mm overjet, and the outer shield of both jaws were flatted, which meant the front wall of lower jaw was strengthened with bumper, termed as lower bumper. A complete dentition model was applied to the study. 150 N occlusal force was applied to each type of appliance and the deformation displacement and the changes in stress was recorded.

**Results:**

The deformation was significant in the incisors regions, especially in the vertical and lateral dimensions. The maximum displacements of 3 mm overjet with step shield group were 7.08 mm (vertical), 3.99 mm (lateral), and 2.90 mm (sagittal), while it decreased to 3.92 mm(vertical), 1.94 mm (lateral), and 1.55 mm (sagittal) in overjet with bumper group. Moreover, the upper molar regions exhibited higher vertical and sagittal displacement in 3 mm overjet with step shield group, which were 3.03 mm (vertical) and 1.99 mm (sagittal), and the bumper design could decrease the maximum displacement to 1.72 mm (vertical) and 0.72 mm (sagittal). In addition, the Von Mises stress of appliances was analyzed, and results indicated that 3 mm overjet with step shield generated higher stress than other groups, with the maximum Von Mises stress was 0.9387 MP, which were 0.5858 and 0.5657 MP in edge to edge group and 3 mm overjet with lower bumper group, respectively.

**Conclusion:**

The prefabricated myofunctional appliances may cause deformation during occlusion. Compared to step shield group, the application of lower bumper exhibited better resistance to occlusal force.

## Background

The prevalence of malocclusion and occlusal traits is common in early mixed dentition. Previous study showed that 79.4% children lived in Shanghai presented occlusal anomalies in mixed dentition [1]. However, in mixed dentition, the deciduous teeth will loose and fall off, which will influence the integrity of dentition and hinder the application of orthodontic appliance on teeth. Therefore, orthodontic treatment often starts after dental transitional period. Even though 2 × 4 appliance could be applied in mixed dentition patients, it mainly aligns the anterior teeth, and shows little regulatory effects on undesirable oral habits and the balance of intraoral and extraoral muscle groups [2, 3]. Harmful oral habits could induce unbalance of maxillofacial muscles, and result in malocclusions [4, 5]. Therefore, it is important to correct the harmful oral habits and balance the maxillofacial muscles in mixed dentition patients [6]. However, the device for dental transitional period is limited in clinic.

Prefabricated myofunctional appliances could guide teeth eruption, improve teeth alignment, correct abnormal functions of muscles, and rectify harmful oral habits, thus enhance the severity of malocclusions, and reduce the difficulties of treatment at the follow up stage [7–15]. Some commercial prefabricated appliance has been applied in mixed dentition to address such problems for a period of time. Prefabricated myofunctional appliance has also been applied to obstructive sleep apnea (OSA). OSA during childhood could induce neuropsychological and cognitive impairment, hypertension, and endocrine disorder. In the treatment of pediatric OSA, surgery was performed first to remove the enlarged tonsils and adenoids. Although surgery could significantly reduce obstruction and clinical symptoms, it still cannot solve all problems, and there was still a large number of patients who needed follow-up treatment. Previous studies found that prefabricated myofunctional appliance could significantly reduce the apnea and hypopnea index (AHI) in children with mild to moderate OSA [16]. Moreover, the application of prefabricated myofunctional appliance after surgery significantly reduced the AHI compared with surgery only group [17]. In addition, most temporomandibular disorder (TMD) patients suffered from temporomandibular joint clicking, pain, difficulty in mouth opening, and even psychological disorders [18]. A correct position of mandible could relief the symptoms of TMD and even improve an athletes’ performance, and occlusal therapy has been considered as an efficient trail for TMD treatment [19, 20]. As an occlusal appliance, prefabricated myofunctional appliance has been applied for the treatment of TMD. Previous multi-centered randomized controlled trails indicated that of prefabricated myofunctional appliance significantly improved the symptoms of TMD, and both short- term and long- term effectiveness of prefabricated myofunctional appliance was in equilibrium with stabilization appliance [18, 19, 21].

Although prefabrication myofunctional appliance has shown positive therapeutic effects in many aspects, the application of prefabricated myofunctional appliance should be carefully evaluated, and strict adherence to indications is necessary. Up till now, the commercial appliance was designed as edge-to-edge, and the outer shield is flattened. However, such a design was not fitful for sagittal skeletal discrepancy, especially skeletal class II malocclusion, which might induce teeth compensatory inclination, and result in difficulties in later guiding mandible advancement. A systematic review compared the effectiveness of prefabricated myofunctional appliance and activator appliance on guiding mandibular advancement of Class II division 1. Results indicated that activator showed better improvement than prefabricated myofunctional appliance. Most important, prefabricated myofunctional appliance treated group exhibited obvious vestibuloversion inclination of mandibular incisors [17]. Considering the sagittal skeletal discrepancy, we recommended to manufact prefabricated myofunctional appliances with different overjet to meet the necessity of different sagittal profile. Furthermore, under occlusal force, the shape deformation of soft silicone functional regulator, both edge-to-edge design and overjet design, was unknown, which was of vital importance as it influenced the torque and tipping of the adjacent teeth.

Even though the overjet design aimed to avoid the compensatory labial inclination of lower anterior teeth, it made the structure at both ends of the orthodontic appliance weaker, which resulted in greater distortion and deformation of the appliance during occlusion. In order to stabilize the anterior and posterior segment of the appliance under occlusion, the lower bumper at lower anterior region was designed to resist deformation. To study the shape changes of prefabricated myofunctional appliance with different overjet and bumper design, we generated three samples of prefabricated myofunctional appliances, including edge-to-edge, 3 mm overjet with step shield, 3 mm overjet with lower bumper strengthened. The deformation displacement and the changes in stress under occlusion force were recorded, which provided references for the design and application of prefabricated myofunctional appliances.

## Methods

**Fig. 1:**
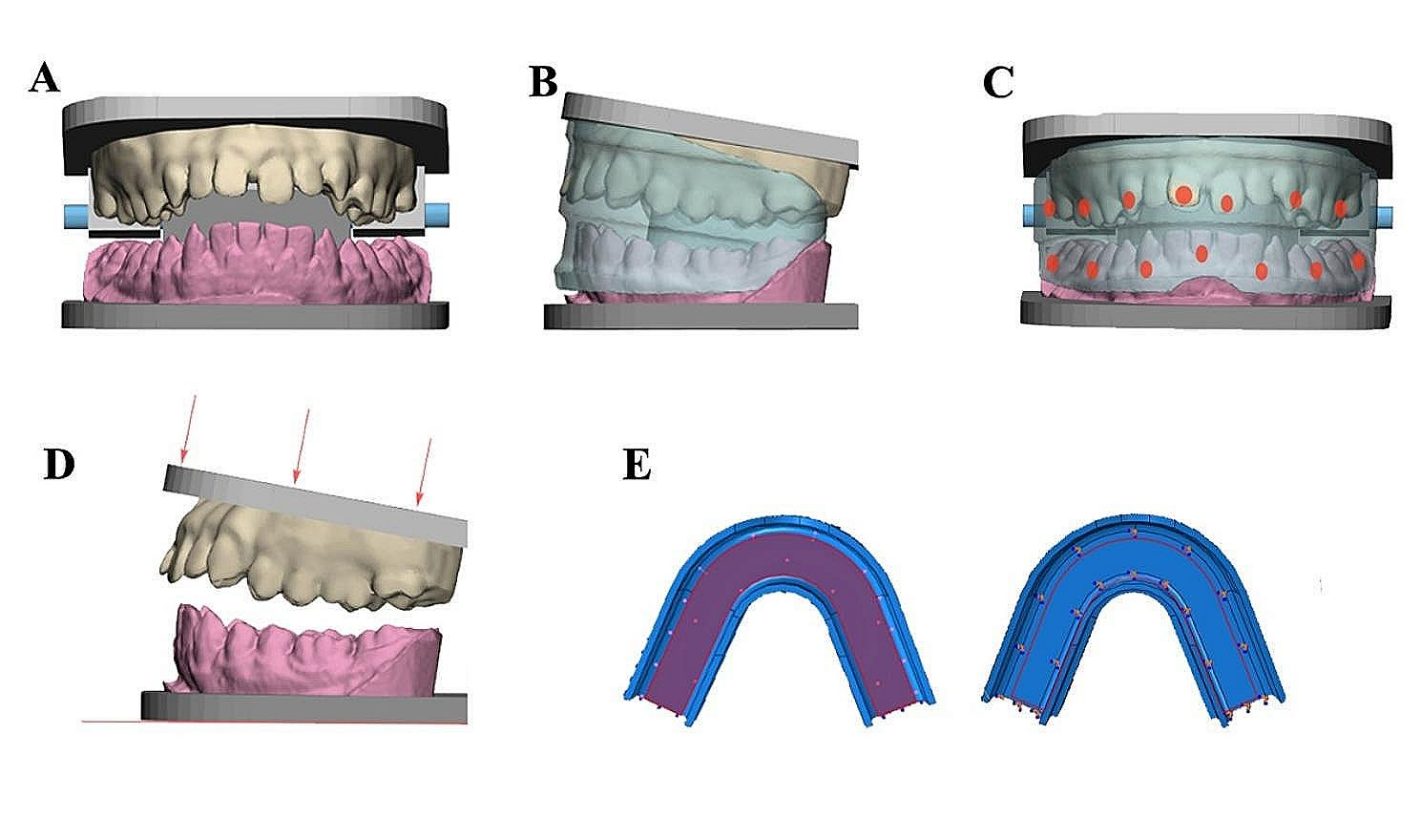
Diagram shows the generation of myofunctional appliance, the pressure gauzes position, and the force loading method. The dentition was scanned and imported in Unigraphics NX 13.0 software (**A**). The myofunctional appliances were designed according to the dentition (**B**). Pressure gauzes were placed on the incisor regions, canine regions, and first molar regions of both jaws (**C**). The force was applied from the upper dentition and the lower dentition was fixed (**D** and **E**)

The subject of this study was a patient who receive orthodontic treatment in the department of orthodontics of Shanghai Ninth Peoples’ Hospital. The selection criteria were deep overbite, fairly aligned dentition, no periodontal disease, and no third molar. The dentition was scanned with 3Shape Trios Oral Scanner (3Shape, Denmark). After scanning, the STL file was then imported into Unigraphics NX 13.0 software (Siemens PLM Software, America), and the arch shape was depicted with four-points reduction method (Fig. 1A).

The lower arch was selected as a reference, and the boundary extended 3 mm toward buccal and labial sides, and 10 mm toward lingual side. This bounary was confirmed as the boundary of the lower occlusal surface of prefabricated myofunctional appliances. Based on the established occlusal regions of the lower dentition, the labial and lingual edge walls of the orthodontic appliance were designed. After the three-dimensional model of the mandibular orthodontic appliance was established, an upper jaw appliance with the same shape and size as the lower jaw appliance was created through mirror operation, and then upper and lower jaws were combined to form an integrated soft appliance (Fig. 1B).

Based on the integrated soft appliance, the lower jaw moved lingually for 3 mm using the deviation function of Unigraphics NX 13.0 software, so that a 3 mm overjet was formed. At the same time, the outer shield of the soft orthodontic appliance also formed a 3 mm sagittal deviation.

On the basis of a soft orthodontic appliance with 3 mm overjet and step outer shield, modifying its our shield parameters to generate an outer bumper until the upper and lower shield was flatted, while maintaining the 3 mm overjet.

All three types of soft orthodontic models were constructed with 50 HA Shore hardness silicone, with a material mass density (RHO) of 1000 kg/m^3^, Young’s modulus (E): 6.10E + 6 Na/m^2^, Poisson’s ratio: 0.49, Shear modulus (room temperature): 1Mpa, Structural damping coefficient (Shore 50 HA): 0.45; Yield strength: 9.24 N/mm^2^ (MPa). The dead-weight and inertia release of the models were ignored in Unigraphics NX 13.0 software. The meshing size was 1.5 mm.

The maxillary anterior teeth were contacted with the inner groove of the bumper of prefabricated myofunctional appliances, and the lower dentition was located 5 mm lingually to the upper dentition. Use a 6 mm x 6 mm rectangle to simulate the occlusal surface of the first molars. The occlusal surface of the upper jaw is loaded with a 150 N occlusal force, and the lower jaw is fixed (Fig. 1D and E).

Pressure gauges have been attached on the orthodontic appliances at the observative points. Simulate the force situation under different conditions and record the force and deformation of different orthodontics via using pressure gauges. The pressure gauges were set at the central incisors, canine, and first molars of both jaws (Fig. 1C).

To quantitatively study the displacement of pressure points, this study established a coordinate system with the width displacement of the dental arch as X, the vertical displacement as Y, and the sagittal displacement as Z.

## Results

### Displacement deformation of three types of prefabricated myofunctional appliances

**Fig. 2:**
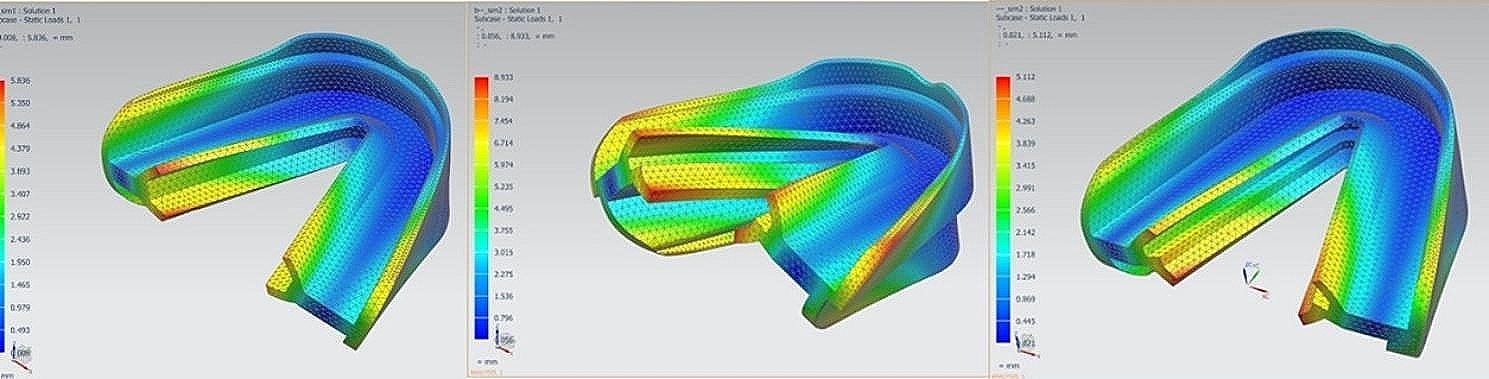
The displacement deformation of three kinds of appliances under occlusion. The left figure exhibited the displacement of Edge-to edge group, the middle figure was 3 mm overjet with step shield group, and the right figure was 3 mm overjet with bumper strengthen group

After 150 N occlusal force was loaded, the displacement of the three types of prefabricated myofunctional appliances were recorded. The results showed that the overall deformation trend of the three types of prefabricated myofunctional appliances were consistent, indicating the bilateral free ends of the prefabricated myofunctional appliances rotation, which increases the buccal crown torque of the upper molars and the lingual crown torque of the lower molars. Additionally, the lingual displacement at the edge of maxillary shield might increase lingual root torque of the maxillary anterior teeth (Fig. 2). In terms of the degree of deformation, the prefabricated myofunctional appliances with 3 mm overjet and step shield (OJ-Step) exhibited the largest overall displacement, while the overall displacement of the edge to edge appliance (ETE) and the 3 mm overjet with lower bumper strengthened (OJ-Bumper) were similar. Results showed that the lateral and vertical displacement of the myofunctional appliances is more obvious than sagittal direction. Moreover, the displacement at incisor and molar regions were higher than canine region.

The quantitative results indicated that the deformation at the upper incisor region was the most prominent. The maximum vertical displacement at upper incisor region was 7.08 mm in OJ-Step group, while it was 5.76 mm and 3.92 mm in ETE group and OJ-Bumper group, respectively. The maximum lateral displacement of OJ-Step group was 3.99 mm, while it was 3.55 mm and 1.94 mm in the ETE group and OJ-Bumper group, respectively. The lower bumper also decreased the sagittal deformation at the edge of upper incisor region from 2.90 mm to 1.55 mm. At the lower incisor region, OJ-Step group showed higher vertical displacement, which was 4.95 mm. However, the lower bumper significantly decreased the displacement to 1.58 mm. The maximum lateral displacement was about 2.5 to 2.6 mm in ETE group and OJ-Bumper group, while it was decreased to 0.60 mm with lower bumper application.

The vertical displacement of the upper left molar region was about 2.46 mm in ETE group. And the parameters were further increased to 3.03 mm in OJ-Step group. The displacement was decreased to 1.72 mm by applying lower bumper to strength the appliance. The maximum sagittal displacement was also decreased from about 1.93 mm to 0.72 mm by lower bumper. The differences of maximum lateral displacement were not significant among three groups. The lower bumper also decreased the vertical displacement of lower left molars from about 2 mm to 0.69 mm, and decreased the lateral displacement from about 3.50 mm to 1.00 mm.

In the upper canine region, the three-dimensional displacement of OJ-Bumper group was significantly decreased compared with OJ-Step group. It is worth noting that the average sagittal displacement of lower bumper group was only 0.09 mm, which was negligible. Since there was no obvious displacement at lower canine region, all three groups of appliances showed little differences at lower canine region. (Table 1).

**Table 1:** The maximum and average displacement amplitude of three kinds of appliances under occlusion at different depression gauzes

	Pressure gauge position	Edge-to-edge						3 mm overjet with step shield						3 mm overjet with lower bumper					
		X	Y	Z	XY	YZ	ZX	X	Y	Z	XY	YZ	ZX	X	Y	Z	XY	YZ	ZX
Maximum Displacement (mm)	Upper Incisor	3.55	5.76	2.67	0.15	0.67	0.03	3.99	7.08	2.90	0.22	0.56	0.20	1.94	3.92	1.55	0.07	0.57	0.07
	Upper Left Canine	1.41	2.75	1.73	0.94	0.22	0.27	2.04	3.27	2.28	1.46	0.74	0.25	0.19	1.73	0.14	1.13	0.04	0.09
	Upper Right Canine	1.97	2.87	1.89	1.05	0.20	0.30	1.25	2.59	1.34	1.41	0.75	0.34	0.23	1.76	0.17	1.16	0.04	0.07
	Upper Left Molar	2.03	2.46	1.93	0.86	0.03	0.16	1.62	3.03	1.99	1.32	0.05	0.12	1.95	1.66	0.72	0.67	0.03	0.05
	Upper Right Molar	1.98	2.41	1.80	0.83	0.06	0.17	1.63	2.94	1.55	1.37	0.06	0.04	1.93	1.72	0.74	0.67	0.01	0.05
	Lower Incisor	2.51	1.70	3.41	0.22	0.54	0.12	2.63	4.95	1.95	0.31	2.31	0.28	0.60	1.58	1.71	0.07	0.24	0.09
	Lower Left Canine	1.43	0.69	1.17	0.76	0.14	0.44	2.58	1.36	1.59	1.30	0.21	0.26	1.53	0.00	0.83	0.91	0.06	0.10
	Lower Right Canine	2.70	1.49	2.33	0.80	0.13	0.19	3.52	1.09	2.48	1.27	0.20	0.23	1.47	0.67	0.75	0.88	0.05	0.13
	Lower Left Molar	2.75	1.70	1.61	0.61	0.05	0.05	3.59	2.07	1.91	1.12	0.04	0.12	1.00	0.69	0.53	0.46	0.02	0.05
	Lower Right Molar	3.29	2.29	2.09	0.63	0.04	0.09	3.92	2.26	2.18	1.16	0.06	0.11	1.09	0.84	0.69	0.40	0.02	0.05
Average Displacement (mm)	Upper Incisor	1.97	4.04	1.22	0.11	0.41	0.03	1.82	4.49	1.30	0.14	0.31	0.11	0.83	2.81	0.58	0.04	0.22	0.04
	Upper Left Canine	0.76	1.95	0.86	0.88	0.10	0.15	0.73	1.95	0.73	1.34	0.37	0.13	0.11	1.58	0.09	1.07	0.02	0.05
	Upper Right Canine	1.00	2.02	0.94	1.00	0.13	0.18	0.64	1.92	0.65	1.30	0.42	0.19	0.11	1.58	0.09	1.12	0.02	0.04
	Upper Left Molar	1.48	1.97	1.51	0.79	0.02	0.12	1.39	2.71	1.57	1.25	0.04	0.04	1.63	1.38	0.54	0.62	0.01	0.02
	Upper Right Molar	1.32	1.84	1.44	0.78	0.04	0.09	1.25	2.63	1.30	1.26	0.03	0.01	1.75	1.50	0.55	0.64	0.01	0.02
	Lower Incisor	1.26	1.22	1.47	0.09	0.50	0.07	1.55	3.61	1.20	0.16	1.33	0.11	0.38	1.05	1.32	0.04	0.09	0.04
	Lower Left Canine	1.14	0.34	0.67	0.69	0.07	0.22	1.74	0.75	0.66	1.23	0.11	0.09	1.29	0.24	0.53	0.86	0.03	0.05
	Lower Right Canine	1.64	0.73	1.18	0.72	0.04	0.11	2.41	0.78	1.14	1.24	0.11	0.10	1.22	0.39	0.46	0.85	0.03	0.05
	Lower Left Molar	2.46	1.47	1.45	0.57	0.02	0.03	3.29	1.71	1.58	1.02	0.03	0.05	0.90	0.63	0.47	0.43	0.01	0.03
	Lower Right Molar	2.64	1.65	1.61	0.58	0.02	0.05	3.59	2.00	1.76	1.06	0.03	0.06	0.82	0.59	0.53	0.38	0.01	0.02

Additionally, considering the depression gauzes were set at the buccal/labial walls of the appliances and the lingual wall of posterior teeth also exhibited obvious deformation, we measured the maximum and minimum displacement amplitude of the lingual wall at the same time. The results revealed that the maximum displacement of OJ-Step group was 8.933 mm, while it was 5.836 mm and 5.112 mm in the ETE group and OJ-Bumper group, respectively. The minimum displacement of OJ-Bumper was 0.05601 mm, while the minimum displacements were 0.007538 and 0.02084 mm in the ETE group and 3 mm overjet with lower bumper strengthened group, respectively (Table 2).

**Table 2:** The maximum and minimum displacement amplitude at the lingual walls of three kinds of appliances under occlusion

Displacement Amplitude(mm)	Edge-to-edge	3 mm overjet with step shield	3 mm overjet with lower bumper strengthened
Maximum	5.836	8.933	5.112
Minimum	0.007538	0.05601	0.02084

Due to the displacement deformation of prefabricated myofunctional appliances while biting, a buccal movement force is exerted on the maxillary posterior teeth and a lingual movement force is exerted on the mandibular posterior teeth collectively. Therefore, the greater overall displacement of the prefabricated myofunctional appliances increases the likelihood of adverse effects on the buccal lingual position of the posterior teeth. Moreover, the higher deformation at the incisor region would increase the labial inclination of anterior teeth.

### Equivalent stresses of three types of prefabricated myofunctional appliances

**Fig. 3:**
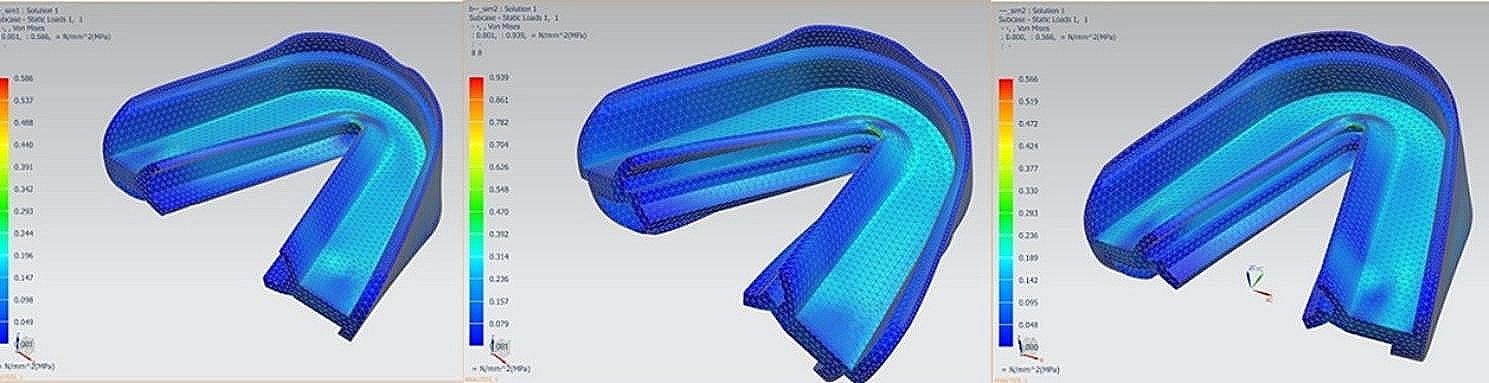
The equivalent stresses cloud map of three kinds of appliances under occlusion. The left figure exhibited the displacement of Edge-to edge group, the middle figure was 3 mm overjet with step shield group, and the right figure was 3 mm overjet with bumper strengthen group

From the equivalent stresses cloud map, it can be found that the Von Mises stress of the prefabricated myofunctional appliances mainly concentrated at the edge of the lingual wall of the anterior teeth, followed by the occlusal area of the teeth. The lowest stress points are all located at the edge of the appliance (Fig. 3). The Von Mises stress of the OJ-Step appliance was significantly higher than that of the other two types of appliances, especially at the incisor and molar regions of both jaws, which is consistent with displacement deformation. The maximum Von Mises stress existed at the upper and lower incisor regions. The Von Mises stress was about 1.6 times higher in IJ-Bumper group than lower bumper group. And the stress was about 1.7 times higher in OJ-Step group than other two groups at the upper molar regions. (Table 3).

**Table 3:** The maximum, minimum, and average Von Mises Stress of three kinds of appliances under occlusion

Pressure Gauge Position	Edge-to-edge			3 mm overjet with step shield			3 mm overjet with lower bumper		
	Minimum	Maximum	Average	Minimum	Maximum	Average	Minimum	Maximum	Average
Upper Incisor	0.16	0.20	0.18	0.21	0.26	0.23	0.14	0.16	0.15
Upper Left Canine	0.12	0.14	0.13	0.17	0.20	0.19	0.16	0.17	0.16
Upper Right Canine	0.13	0.15	0.14	0.18	0.19	0.18	0.16	0.17	0.17
Upper Left Molar	0.09	0.11	0.10	0.16	0.17	0.17	0.08	0.11	0.10
Upper Right Molar	0.08	0.10	0.10	0.16	0.19	0.17	0.10	0.11	0.10
Lower Incisor	0.06	0.14	0.10	0.20	0.31	0.27	0.19	0.25	0.22
Lower Left Canine	0.10	0.12	0.11	0.19	0.21	0.20	0.13	0.14	0.13
Lower Right Canine	0.11	0.11	0.11	0.20	0.21	0.20	0.13	0.14	0.14
Lower Left Molar	0.08	0.10	0.09	0.14	0.18	0.16	0.05	0.06	0.06
Lower Right Molar	0.09	0.10	0.10	0.16	0.18	0.17	0.04	0.05	0.05

The maximum Von Mises stress at the lingual walls of OJ-Step was 0.9387 N/mm^2^ (MPa), while the maximum Von Mises stresses were 0.5858 and 0.5657 N/mm^2^ (MPa) in the ETE group and OJ-Bumper group, respectively. The minimum Von Mises stress at the lingual walls of OJ-Step was 0.00121 N/mm^2^ (MPa), while the minimum Von Mises stresses were 0.0006814 and 0.0004228 N/mm^2^ (MPa) in the ETE group and OJ-Bumper group, respectively (Table 4).

**Table 4:** The maximum and minimum Von Mises Stress at the lingual walls of three kinds of appliances under occlusion

Von Mises Stress(*N*/mm^^2^(MP))	Edge-to-edge	3 mm overjet with step shield	3 mm overjet with lower bumper strengthened
Maximum	0.5858	0.9387	0.5657
Minimum	0.0006814	0.00121	0.0004228

## Discussion

During the mixed dentition period, the maxillofacial bones, muscles and arch dentition of children enter key stage of development. Harmful oral habits are non-negligible causes of malformation in preschool children. Therefore, early intervention with appropriate appliances can break harmful oral habits, allocate dentition space, and improve the severity of malocclusion to a certain extent [6]. Moreover, improvement of malocclusion is also of vital importance for the establishment of children’s mental health [22–24]. Mandibular retrusion and deep overbite are common malocclusion in mixed dentition. However, the device for dental transitional period is limit in clinic. The prefabricated myofunctional appliances could guide teeth eruption, improve teeth alignment, correct functions of muscles, and correct harmful oral habits [7–15]. Up till now, the commercial prefabricated myofunctional appliances are edge to edge design, which might be applied in patients without severe sagittal incongruous. However, for severe mandibular retrusion patients, the edge to edge design might induce unfavorable labial inclination of lower anterior teeth. Therefore, the application of prefabricated myofunctional appliances with overjet is essential. The shape displacement of prefabricated myofunctional appliances could influence the teeth movement and treatment outcomes. However, the study on the shape displacement and stress distribution is lacking. The current study established edge to edge model and 3 mm overjet appliance. Moreover, the 3 mm overjet appliances have also been divided into step shield and lower bumper strengthened to observe the shape displacement and equivalent stress.

By establishing a finite element model of prefabricated myofunctional appliances loaded with 150 N force, the current study indicated that overbite with step bumper design could induce more obvious displacement of soft appliance and greater unfavorable force on teeth. The displacement of the shield will load on the adjacent teeth, and generate abnormal external forces, which increase the lingual root torque of upper anterior teeth and buccal crown torque of lower anterior teeth. Although the overjet decreased at the same time, the unfavorable buccal inclination of lower anterior teeth would hinder the mandibular advancement at the later stage. Previous clinical study also exhibited higher vestibular inclination of lower incisors, which could be ascribed to the shape deformation of prefabricated myofunctional appliance under occlusal force [17]. In transverse dimension, prefabricated myofunctional appliance could generate maxillary expansion effect. However, recent comparative study found that rapid maxillary expansion with hyrax appliance significantly improved the width of dental arch, with 55.6% skeletal change and 44.4% dental change. Additionally, prefabricated myofunctional appliance exhibited higher dental changes (∼ 51.9%) [25]. In the current study, torsion of the posterior aligner appliance tilted the lingual crown torque of lower posterior teeth and buccal crown torque of upper posterior teeth, increased the coverage of the posterior teeth, which may eventually affect the occlusal stability of the posterior teeth. The result explained the phenomenon of higher dental effects in arch expansion. Compared to edge to edge design group, OJ-Bumper showed less maximum displacement, and the difference between the maximum and minimum displacement of OJ-Bumper was less than edge to edge group, indicating that the overall shape of the appliance with lower bumper strengthened was relatively small, and the shape of the appliance was more stable when occlusal force was loaded, which is beneficial for the prediction and controlling of the overall dentition position. In addition to maxillary expansion, prefabricated myofunctional appliance could also be used for reposition bilaterally condyle to symmetric position to solve unilateral scissor bite [26, 27]. Although previous literatures showed more dental effects of prefabricated myofunctional appliance compared with traditional fixed or removable appliances, the prefabricated myofunctional appliance could solve transverse, sagittal, and vertical problems simultaneously. In order to overcome the tooth tilting caused by the deformation of soft silicone appliances, this study advocated the selection of appliances with overjet design. At the same time, to combat shape deformation, a lower bumper was used, which overcome unfavorable teeth inclination both at sagittal and transversa dimensions.

The stress analysis of the three appliances showed that 3 mm overjet with step shield would have larger Von Mises stress distribution, and the stress magnitude was significantly greater than that of other appliances, which might induce fracture from the inside due to excessive stress on the appliance during occlusion. The Von Mises stress distribution range and stress ratio of OJ-Bumper appliance were slightly lower than those of edge to edge appliance, indicating lower internal stress and more stable overall shape, which was conducive to the stable conduction of orthodontic force.

There were still some limitations in this study. First of all, the current study only used a complete dentition model for research. However, in clinical practice, malocclusion presented with a variety of clinical manifestations, e.g. alignment problem, skeletal Class II or III, deep overbite or open bite, crossbite and so on. Different occlusions resulted in different force loading modes and shape deformations. Therefore, the shape deformation and forces of different malocclusions should be investigated in the future. Moreover, different attachment and appliance design should be applied to solve different clinical problems. Second, due to the different rotation radius of each person’s mandible, as well as the occurrence of abnormal mandibular movement trajectories due to temporomandibular joint disorder or muscle dysfunction, the initial loading position, stress concentration position, and shape deformation of appliance might be different. Third, the loading direction of this study was fixed, which was complex during mastication.

## Conclusion

Based on the findings of this study, the following conclusions can be made:

1. Under occlusal force, the traditional prefabricated myofunctional appliances and OJ-Step design caused obvious shape deformation. This was mainly manifested as inducing unfavorable labial inclination of lower anterior teeth, and higher coverage of posterior teeth, which increased the risk of alveolar bone dehiscence and fenestration.
2. OJ-Step induces higher Von Mises stress than OJ-Bumper group and ETE group, making it prone to breakage during occlusion.
3. Based on the current study, we suggested that clinical practitioners implement staged sagittal reposition when dealing with severe sagittal discrepancy. Moreover, lower bumper was recommended as it enhanced deformation resistance in both sagittal and transversal dimensions.

## Data Availability

The data used and/or analyzed during the current study are available from the corresponding author on reasonable request.

## Abbreviations

ETE: Edge to edge
OJ-Step: 3 mm overjet with step shield
OJ-Bumper: 3 mm overjet with lower bumper strengthened
*OSA*: Obstructive sleep apnea
TMD: Temporomandibular disorder
AHI: Apnea hyponea index
